# Differences in the Effectiveness of Uridine and *Liriope platyphylla* L. Between Complement Component 3 Deficiency- and Loperamide-Induced Constipation May Be Associated with the Alternative Regulation of the Cyclic Adenosine Monophosphate Downstream Signaling Pathway

**DOI:** 10.3390/ph18091289

**Published:** 2025-08-28

**Authors:** Hee Jin Song, Eun Seo Park, Ji Eun Kim, Ayun Seol, Su Jeong Lim, Su Ha Wang, Ye Ryeong Kim, Ye Eun Ryu, So Hae Park, Jumin Park, Hyun Gu Kang, Dae Youn Hwang

**Affiliations:** 1 Department of Biomaterials Science (BK21 FOUR Program), College of Natural Resources and Life Science/Life and Industry Convergence Research Institute, Pusan National University, Miryang 50463, Republic of Korea; hejin1544@naver.com (H.J.S.); geg9393@naver.com (E.S.P.); prettyjiunx@naver.com (J.E.K.); a990609@naver.com (A.S.); soojl1315@naver.com (S.J.L.); dhkdtngk@naver.com (S.H.W.); yr2232@naver.com (Y.R.K.); 5chdtkdlqsl@naver.com (Y.E.R.); sohaehw@pusan.ac.kr (S.H.P.); 2 Department of Food Science and Nutrition, College of Human Ecology, Pusan National University, Busan 46241, Republic of Korea; mminv8@naver.com; 3 Department of Veterinary theriogenology, College of Veterinary Medicine, Chungbuk National University, Cheongju 28644, Republic of Korea; kang6467@cbu.ac.kr

**Keywords:** complement C3, loperamide, constipation, AELP, uridine, cAMP

## Abstract

**Background/Objectives**: Constipation can be induced in animal models through various factors such as loperamide (Lop) or complement component 3 (C3) deficiency. The effectiveness of therapeutic agents in the clinical management of constipation has been primarily evaluated within only one model, but between-model comparisons have not been performed so far. Therefore, we investigated whether the effectiveness of the laxative drugs for the clinical management is related to etiological factors. **Methods**: The changes in the key parameters for defecation were compared between C3 knockout (KO) mice with C3-deficiency-induced constipation and ICR mice with Lop-induced constipation after the oral administration of Uridine (Urd) and aqueous extract of *Liriope platyphylla* L. (AELP). **Results**: Similar effectiveness of Urd and AELP were detected on the stool frequency, intestinal epithelial barrier structure, and mucin secretion in both models. However, other parameters (namely gastrointestinal (GI) transit, water retention, and enteric nervous system (ENS) structure and function) showed higher effectiveness in C3 KO mice than in the Lop-induced model. Only the effectiveness of the two therapeutic agents on the histological structure of the mid-colon was greater in the Lop-induced mice model compared to the C3 KO mice model. Furthermore, these differences in the therapeutic effectiveness of Urd and AELP were partially reflected in alterations in the cyclic adenosine monophosphate (cAMP) downstream signaling pathway. **Conclusions**: The results suggest that the therapeutic effectiveness of Urd and AELP is sensitive to C3-deficiency-induced constipation and these differences may be linked to the alternative regulation of the cAMP downstream signaling.

## 1. Introduction

Constipation is a common gastrointestinal (GI) condition characterized by irregular bowel movements, hard and dry stools, feeling of incomplete bowel evacuation, and difficulty and pain during defecation although it has been classified as acute and chronic [1,2,3]. The etiology of this disorder is considered very important in the clinical efficacy management of a constipation patient because it is generally induced by a variety of causes [4,5]. Primary causes are defined as a decrease in intestinal function without any specific disease and includes irritable bowel syndrome with constipation (IBS-C), slow transit constipation, and functional defecation disorders [6]. But, secondary causes are other diseases or medications including opioids, certain antidepressants, calcium channel blockers, and iron supplements [6]. Therefore, the best clinical management for each patient is determined based on the symptoms and causes of constipation and the patient’s lifestyle [5]. Many comparative data on the effectiveness of the agents based on the cause of constipation should be sufficiently provided to clinicians.

The efficacy and action mechanism of various clinical management used for the management of constipation are investigated through animal models. Among them, the rat model of loperamide (Lop)-induced constipation is the most commonly used in pre-clinical studies because it exhibits a distinct phenotype for constipation including a decrease in stool frequency, mucin secretion, GI motility, and disruption of histopathological structures [7,8]. Several other chemicals, including clonidine, morphine, opioid receptor antagonists, such as naltrexone and naloxone, clozapine, and carbon, are also known to cause constipation [9,10,11,12,13]. Animal models with constipation induced by these agents have been used to evaluate the efficacy of therapeutic agents in alleviating constipation-related symptoms and to study the mechanisms of action [14,15,16]. It has been recently reported that complement component 3 (C3) deficiency can cause a novel type of constipation in the C3 knockout (KO) mice, and these symptoms were significantly relieved by the administration of the aqueous extract of *Liriope platyphylla* L. (AELP) and Uridine (Urd) [17,18]. However, previous studies have not provided scientific evidence for the differences in effectiveness of the agents used to treat constipation based on their causes, and their efficacy analyses have been focused on only a few animal models.

Meanwhile, cyclic adenosine monophosphate (cAMP) is a well-known signaling pathway that regulates various cellular mechanisms, including growth, differentiation, and gene transcription, and is considered a potential target for several chronic diseases [19]. This molecule is involved in the induction mechanism of disease in a few constipation models. In the Lop-induced constipation model, the cAMP signaling pathway is inhibited by binding Lop and the opioid receptors, which subsequently increases the absorption of ions such as moisture and electrolytes and decreases the relaxation of smooth muscles [20]. In addition, the C3 protein is associated with the cAMP signaling pathway. When C3a binds to its receptor, the cAMP levels are reduced, and this response can enhance the capacity of dendritic cells for antigen uptake and T-cell stimulation [21]. The above responses may be disrupted during C3-deficiency-induced constipation. Therefore, cAMP can be considered an action mechanism commonly involved in the two models of constipation used in our study, although its exact mechanism has not been verified in C3 KO mice. In addition, Urd and AELP were specifically selected as a single chemical drug and a natural product with laxative effects, respectively, because their therapeutic effects were successfully demonstrated in the Sprague-Dawley (SD) rat model with Lop-induced constipation and the C3 KO mice model [18,22,23].

In this study, we investigated whether the differences observed in the effectiveness of various agents used to treat constipation can be linked to the cause of constipation. For this, therapeutic-effects-related parameters, including stool frequency, GI transit, histo-pathological features, mucin secretion ability, water retention capacity, and enteric nerv-ous system (ENS) structure and function, were compared between the C3 KO mice and Lop-induced mice models after administration of Urd and AELP.

## 2. Results

### 2.1. Laxative Effects of Urd and AELP in Mice with C3 KO and Lop-Induced Constipation: Comparison of Stool Parameters

**Figure 1:**
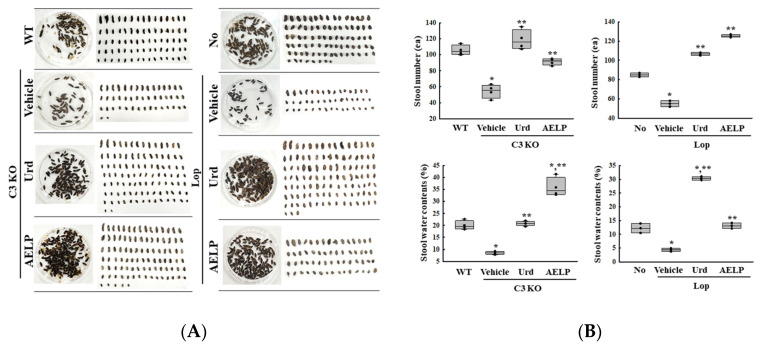
Stool parameters analyses of C3 KO and Lop-induced constipation mice model. (**A**) Morphology of stools. (**B**) Level of stools-related parameters. * indicated that the *p*-value is 0.05 or less compared to the WT group. ** indicated that the *p*-value is 0.05 or less compared to the Vehicle-treated C3 KO group. Abbreviations: C3 KO, Complement component 3 knockout; Urd, Uridine; AELP, Aqueous extract of *Liriope platyphylla* L.; WT, Wild type.

First, we investigated whether there was a difference in the therapeutic effectiveness of Urd and AELP in altering the stool parameters between the C3-deficiency- and Lop-induced constipation models. The changes in the stool number and water content were compared between the two groups after the administration of Urd and AELP. The effects of Urd on the stool number were higher at 37.6% in the C3 KO mice than in the Lop-injected mice, while those of AELP were higher at 78% in the Lop-injected mice and C3 KO mice (Figure 1 and Table 1). However, a reverse pattern was detected in the stool water contents. Higher effects of Urd were detected in Lop-treated mice (320%) than those of AELP in the C3 KO mice (58%) (Figure 1 and Table 1). These results suggest that the therapeutic effectiveness of Urd and AELP on the inhibition of stool parameters is less linked to the specific constipation models with different induction mechanisms.

### 2.2. Laxative Effects of Urd and AELP in the C3 KO and Lop-Induced Constipation Models: Comparison of GI Transit

**Figure 2:**
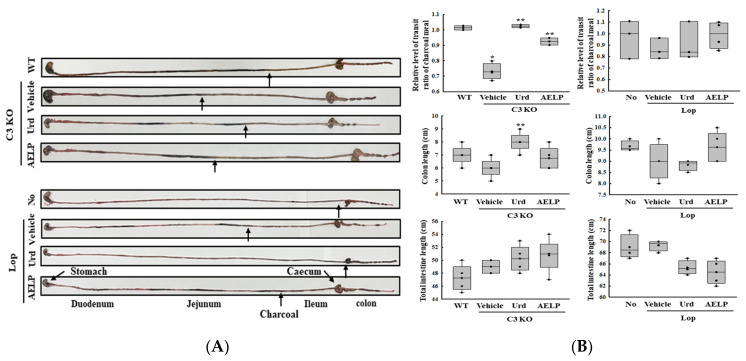
GI transit and length of C3 KO and Lop-induced constipation models. (**A**) Morphology of the GI tract. (**B**) Transit ratio of charcoal meal, colon length, and total intestine length. * indicated that the *p*-value is 0.05 or less compared to the WT group. ** indicated that the *p*-value is 0.05 or less compared to the Vehicle-treated C3 KO group. Abbreviations: C3 KO, Complement component 3 knockout; WT, Wild type; Urd, Uridine; AELP, Aqueous extract of *Liriope platyphylla* L.; GI, Gastrointestinal; Lop, Loperamide.

To investigate whether there was a difference in the therapeutic effectiveness of Urd and AELP between the C3 KO and Lop-induced constipation models in disrupting the GI transit, alterations in the charcoal meal transit ratio, GI length, and colon length were compared. The improvement in the charcoal transit ratio and colon length in the Urd-treated groups was 5.6 times and 19.3 times greater in the C3 KO model than in the Lop-induced constipation model although the total GI length remained constant (Figure 2 and Table 2). However, there was no difference in the therapeutic effectiveness of AELP between C3 KO and Lop-induced constipation models on the three parameters outlined above (Figure 2 and Table 2). These results suggest that the therapeutic effectiveness of Urd on the GI transit is more specific in the C3 KO model than in the Lop-induced constipation model.

### 2.3. Laxative Effects of Urd and AELP in the C3 KO and Lop-Induced Constipation Models: Comparison of the Histopathological Structure of the Mid-Colon

**Figure 3:**
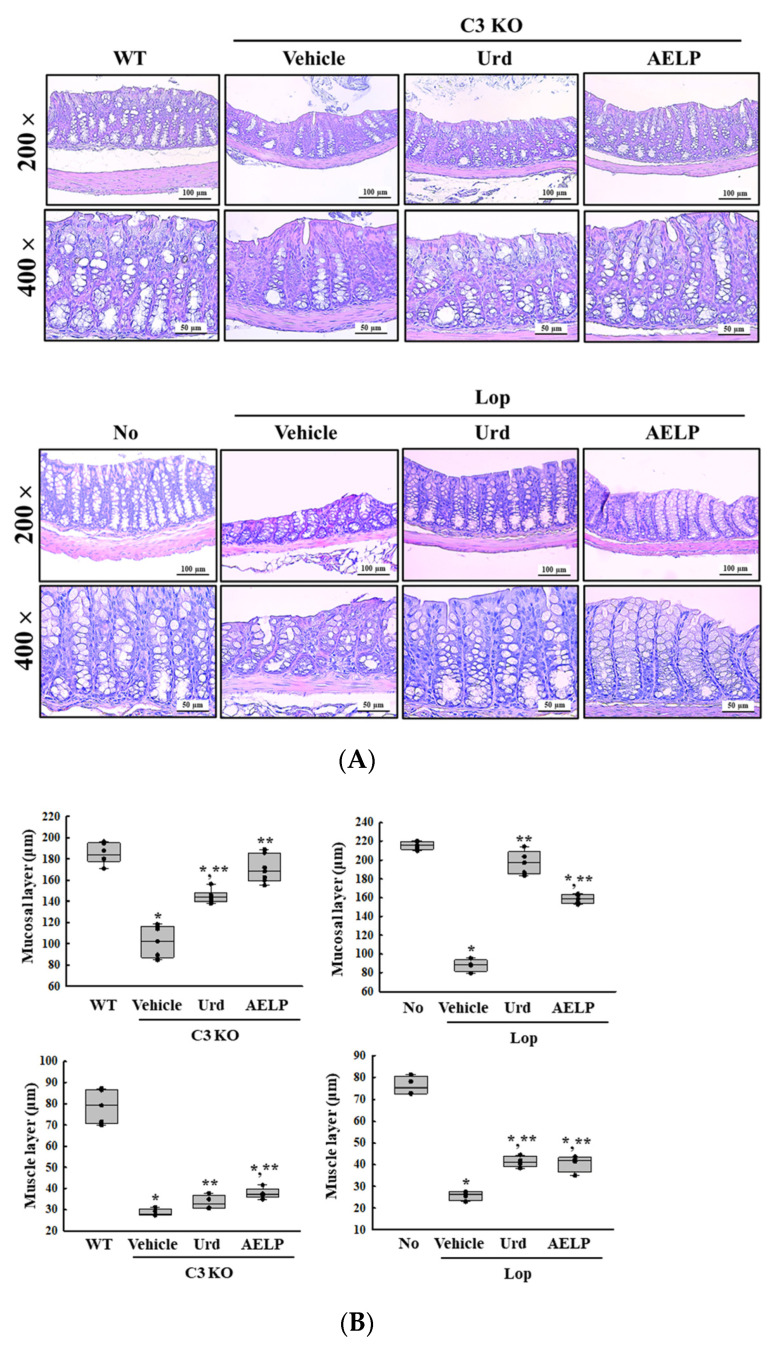
Histopathology of the mid-colon in C3 KO and Lop-induced constipation mice models. (**A**) Images of H&E-stained mid-colon section. (**B**) Thickness of mucosal and muscle layer in mid-colon. The H&E-stained tissue sections were prepared from mid-colons of three to five mice per group, and the pathological factors were analyzed twice for each stained tissue. * indicated that the *p*-value is 0.05 or less compared to the WT group. ** indicated that the *p*-value is 0.05 or less compared to the Vehicle-treated C3 KO group. Abbreviations: C3 KO, Complement component 3 knockout; Urd, Uridine; AELP, Aqueous extract of *Liriope platyphylla* L.; H&E, Hematoxylin and Eosin; Lop, Loperamide.

Next, we investigated whether there was a difference in the therapeutic effectiveness of Urd and AELP between the C3 KO and Lop-induced constipation models in restoring the disrupted histopathological structures of the mid-colon. To achieve this, alterations in the mucosal layer and muscle layer of the hematoxylin and eosin (H&E)-stained mid-colon section were compared in the two groups after the administration of Urd and AELP. The beneficial effects of Urd on the thickness of the mucosal and muscle layers were 1.95 times and 2.2 times greater in the Lop-induced constipation model than in the C3 KO model (Figure 3 and Table 3). Also, a similar pattern was detected after AELP treatment although the improvement was lower than that of Urd. The improved effects of AELP in the Lop-induced model was greater by 66% in only muscle layer (Figure 3 and Table 3). Therefore, the above results suggest that the therapeutic effectiveness of Urd and AELP in restoring the histopathological structure of the mid-colon is higher in the Lop-induced constipation model than in the C3 KO model.

### 2.4. Laxative Effects of Urd and AELP in the C3 KO and Lop-Induced Constipation Models: Comparison of the Intestinal Epithelial Barrier Structure

**Figure 4:**
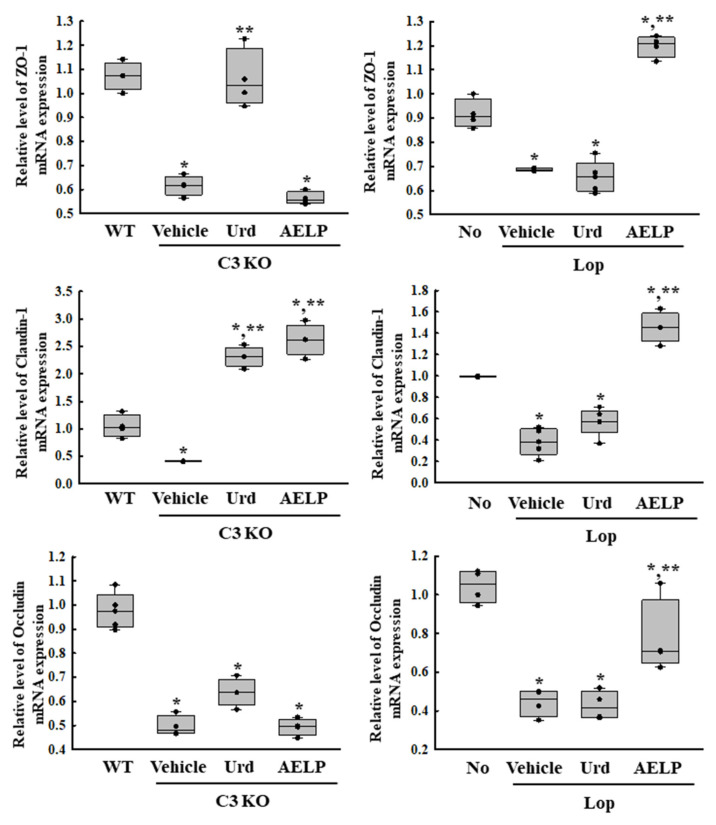
Expression of the junctional complexes in C3 KO and Lop-induced constipation models. Transcriptional level of tight junctional complexes. * indicated that the *p*-value is 0.05 or less compared to the WT group. ** indicated that the *p*-value is 0.05 or less compared to the Vehicle-treated C3 KO group. Abbreviations: C3 KO, Complement component 3 knockout; Urd, Uridine; AELP, Aqueous extract of *Liriope platyphylla* L.; WT, Wild type; Lop, Loperamide; ZO-1, Zonula Occludens-1.

To investigate whether there was a difference in the therapeutic effectiveness of Urd and AELP between the C3-deficient and Lop-induced constipation models in restoring the disrupted intestinal epithelial barrier structure, alterations in the expression of the regulatory proteins for the tight junction (TJ) were compared after administration of Urd and AELP. Among the three regulators, there was increased expression of Claudin-1 in the C3 KO model compared to the Lop-induced constipation model (Figure 4 and Table 4). The expression levels of Occludin were higher only in the AELP-treated Lop-induced constipation model, while the levels were maintained constant in other groups (Figure 4 and Table 4). Also, significant differences were observed in the expression of Zonula Occludens-1 (ZO-1) between the C3 KO and Lop-induced constipation models. Urd effects are greater by 18.8 times in C3 KO mice, but AELP effects are greater by 10.4 times in Lop-induced mice than C3 KO mice (Figure 4). The above results show that the therapeutic effectiveness of Urd and AELP on the downregulation of TJ regulators make no significance difference between C3 KO and Lop-treated mice.

### 2.5. Efficacy of Urd and AELP in the C3 KO and Lop-Induced Constipation Models: Comparison of Mucin Secretion

**Figure 5:**
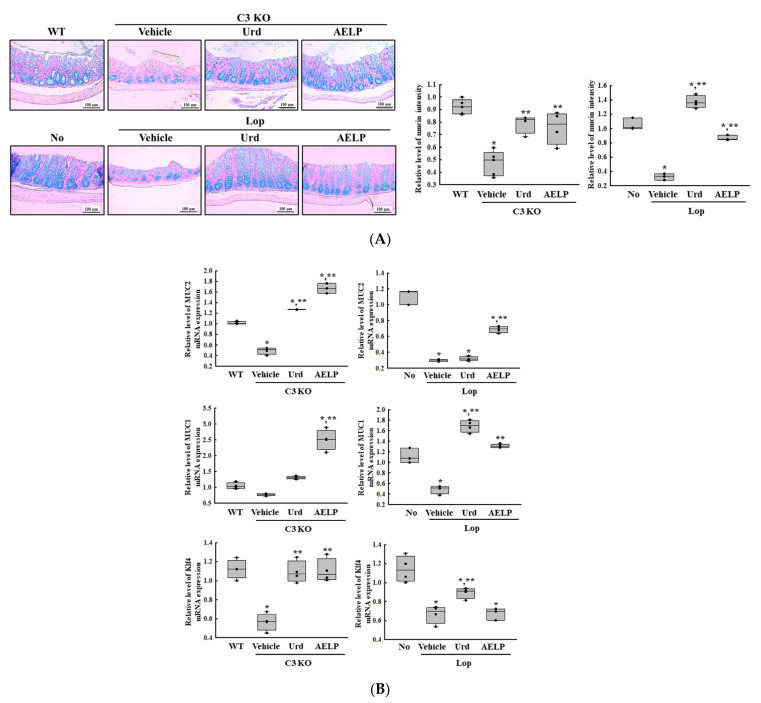
Mucin regulation in C3 KO and Lop-induced constipation models. (**A**) Images of mucin in the Alcian-blue-stained mid-colon section. The blue color density that represented the mucin level in the mid-colon was detected at 200 × magnification. (**B**) Transcription level of MUC-related genes. * indicated that the *p*-value is 0.05 or less compared to the WT group. ** indicated that the *p*-value is 0.05 or less compared to the Vehicle-treated C3 KO group. Abbreviations: C3 KO, Complement component 3 knockout; Urd, Uridine; AELP, Aqueous extract of *Liriope platyphylla* L.; WT, wild type; MUC, mucin; Klf4, Krüppel-like factor 4.

To investigate whether there was a difference between the C3-deficient and Lop-induced constipation models in the therapeutic effectiveness of Urd and AELP against the dysregulation of mucin secretion, alterations in the expression and regulation of the mucin were compared in the two models after administration of Urd and AELP. Among the four factors investigated, the therapeutic effectiveness of Urd and AELP on mucin (MUC)2 and Krüppel-like factor 4 (Klf4) transcription were higher in the C3 KO than in the Lop-induced constipation models, while the effects on the decrease in mucin intensity and MUC1 transcription were greater in the Lop-induced constipation model than in the C3 KO model (Figure 5 and Table 5). Especially, the highest effectiveness (104.6 times) was detected in the Klf4 transcription level in the AELP-treated C3 KO mice, followed by MUC2 transcription in the Urd-treated C3 KO mice (25 times) and mucin intensity in the Urd-treated Lop-induced mice (4.2 times) (Figure 5 and Table 5). These results suggest that the therapeutic effectiveness of Urd and AELP on the secretion of mucin may be closely associated with the Lop-induced constipation model, but the same effects on the transcriptional regulation of the MUC gene may be linked to the C3 KO mice.

### 2.6. Comparison of Water Retention Capacity in the C3 KO and Lop-Treated Mice Due to the Laxative Effects of Urd and AELP

**Figure 6:**
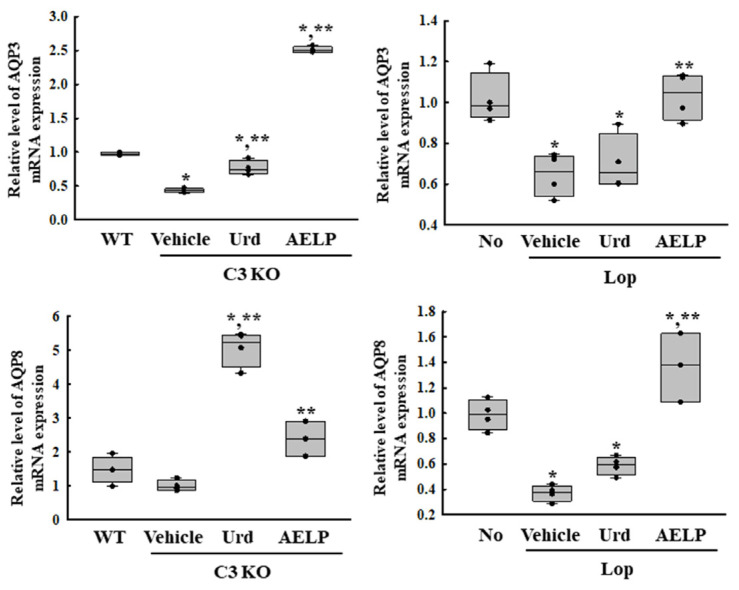
Expression of AQPs in C3 KO and Lop-induced constipation models. Transcriptional level of AQP3 and AQP8. * indicated that the *p*-value is 0.05 or less compared to the WT group. ** indicated that the *p*-value is 0.05 or less compared to the Vehicle-treated C3 KO group. Abbreviations: C3 KO, Complement component 3 knockout; Urd, Uridine; AELP, Aqueous extract of *Liriope platyphylla* L.; AQP, Aquaporins; WT, Wild type; Lop, Loperamide.

To investigate whether there was a difference between the C3-deficient and Lop-induced constipation models in the therapeutic effectiveness of Urd and AELP in improving water retention capacity, alterations in the transcription of aquaporin (AQPs) genes were compared in the two models after administration of Urd and AELP. The transcription levels of AQP3 and AQP8 genes were decreased in both constipation models treated with the Vehicle. However, these levels were remarkably increased in both models after the administration of Urd and AELP (Figure 6 and Table 6). Treatment with Urd increased the transcription of AQP3 7.4 times and 5.8 times for AQP8 in the C3 KO model compared to the Lop-induced constipation model. The improvement effects of Urd and AELP on the downregulation of AQP3 transcription were greater in AELP-treated C3 KO mice than the Lop-induced mice. But these differences were greater in Urd-treated C3 KO mice and AELP-treated Lop-induced mice (Figure 6 and Table 6). Therefore, the above results show that the therapeutic effectiveness of Urd on the downregulation of AQP transcription may be closely associated with only the C3 KO model although these of AELP may similarly link to both models.

### 2.7. Efficacy of Urd and AELP on the ENS Structure and Function of the C3 KO and Lop-Induced Constipation Models

**Figure 7:**
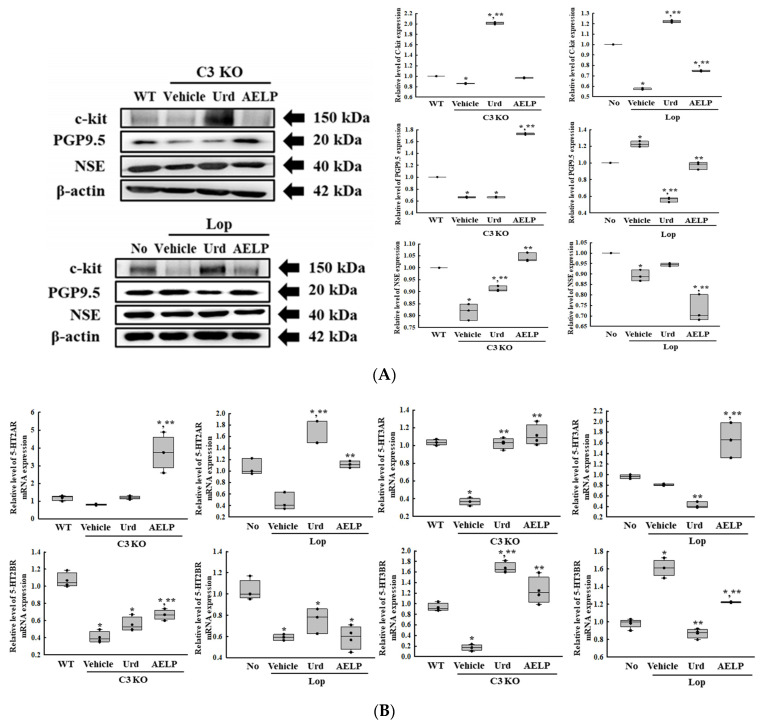

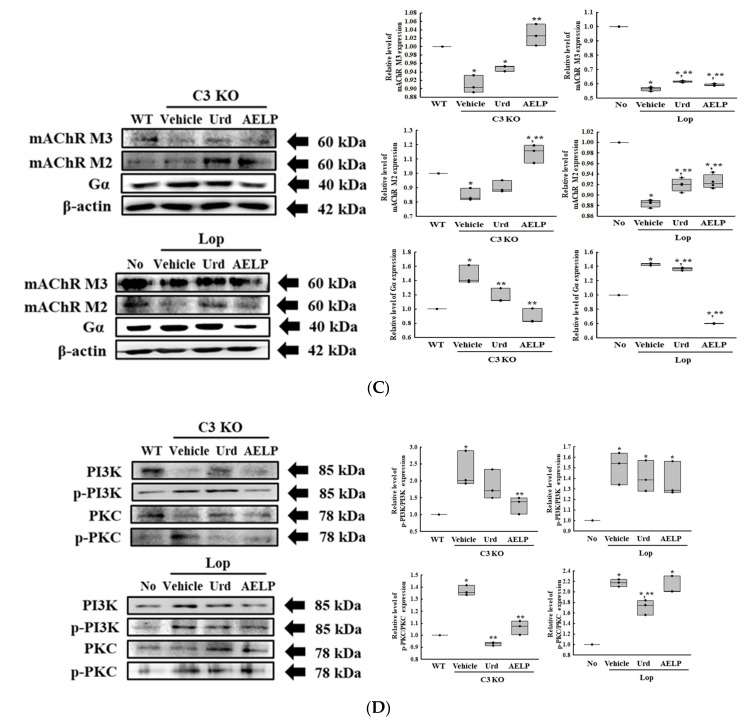
Analyses for ENS-related markers between C3 KO and Lop-induced mice models. (**A**) Expression levels of C-kit, NSE, and PGP9.5. (**B**) Expression of 5-HT receptors. (**C**) Expression level of mAChRs. (**D**) Expression levels of key members of the mAChRs downstream signaling pathway. * indicated that the *p*-value is 0.05 or less compared to the WT group. ** indicated that the *p*-value is 0.05 or less compared to the Vehicle-treated C3 KO group. Abbreviations: C3 KO, Complement component 3 knockout; WT, Wild type; Urd, Uridine; AELP, Aqueous extract of *Liriope platyphylla* L.; C-kit, Receptor protein kinase kit; NSE, Neuron-specific enolase; PGP9.5, Protein gene product 9.5; 5-HT, 5-hydroxytryptamine; mAChRs, Muscarinic acetylcholine receptors; ENS, Enteric nervous system; Lop, Loperamide.

To investigate whether there was a difference between the C3-deficient and Lop-induced constipation models in the therapeutic effectiveness of Urd and AELP in restoring the structural abnormality and dysfunction of the ENS, alterations in the composition of neuronal cells and the excitatory and inhibitory function of the ENS were compared in both models after administration of Urd and AELP. The expression level of markers for the neuronal cells and interstitial cells of Cajal (ICC) was remarkably upregulated in both models after treatment with Urd and AELP, while it decreased in the control group. Treatment with Urd and AELP significantly inhibited receptor protein kinase kit (c-Kit), protein gene product 9.5 (PGP9.5), and neuron-specific enolase (NSE) expressions in the C3 KO model than in the Lop-induced constipation model (Figure 7A and Table 7). Also, Urd and AELP treatments were equally effective with respect to the excitatory function of the ENS. The expression level of four 5-hydroxytryptamine (5-HT) receptors was increased in both models after treatment with Urd and AELP. However, most enhancement effects of Urd and AELP in the downregulation of 5-HT receptor transcription level were greater in the C3 KO mice than those in the Lop-induced mice (Figure 7B and Table 7). Furthermore, there were differences in effectiveness on the parameters for the inhibitory function of the ENS. The downregulation of muscarinic acetylcholine receptors (mAChRs) was significantly increased in both models after the administration of Urd and AELP. The effectiveness of the Urd treatment was higher in the Lop-induced constipation model compared to the C3 KO model. However, the effectiveness of the AELP treatment was greater in the C3 KO model than in the Lop-induced constipation model (Figure 7C and Table 7). Finally, the level of most factors involved in the downstream signaling pathway of the mAChRs were highly effective in the Lop-induced mice regardless of the therapeutics administrated (Figure 7D and Table 7). Therefore, the above results show that the therapeutic effectiveness of Urd and AELP on the structural abnormality and dysfunction of the ENS may be more tightly linked to the C3 KO model than the Lop-induced constipation model.

### 2.8. Identifying the Cause of the Difference in the Therapeutic Effectiveness of Urd and AELP in the C3 KO and Lop-Induced Constipation Models

**Figure 8:**
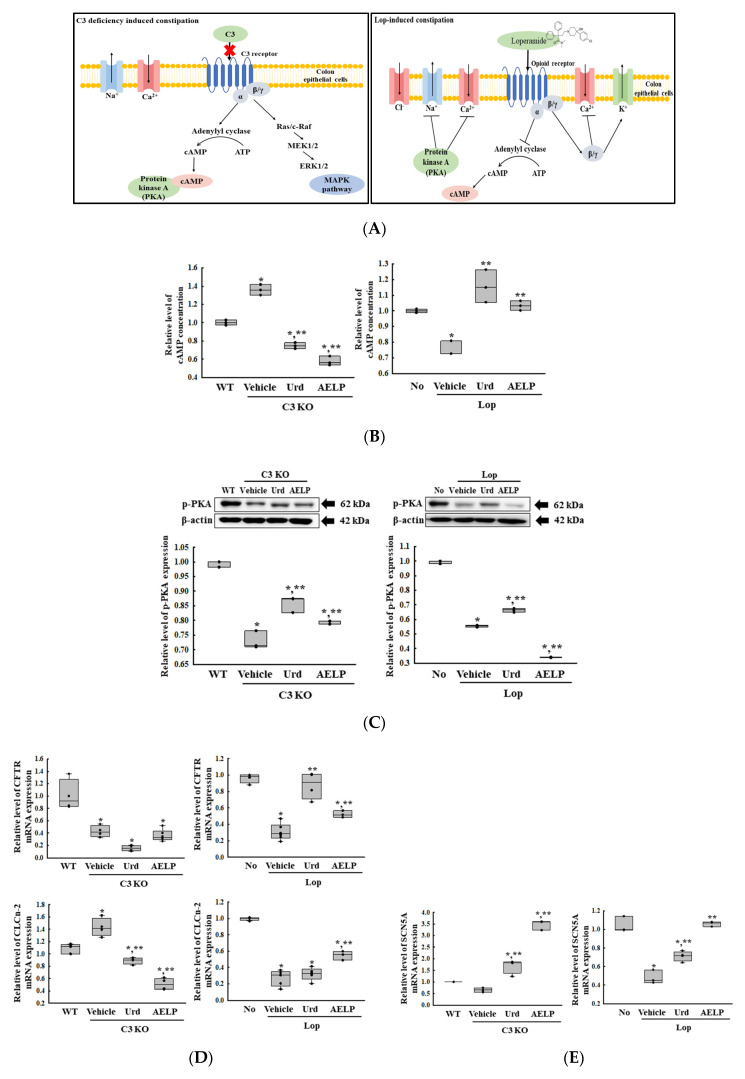

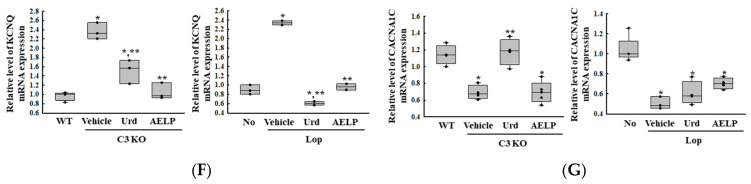
cAMP downstream signaling pathway between C3 KO and Lop-induced constipation models. (**A**) Mechanism for cAMP signaling pathway. The figure was created using Microsoft PowerPoint. (**B**) Concentration of cAMP. (**C**) Protein level of p-PKA. (**D**) Transcription level of CFTR and CLCN2 for Cl ion channel. (**E**) Transcription level of SCN5A for Na ion channel. (**F**) Transcription level of KCNQ for K ion channel. (**G**) Transcription level of CACNA1C for Ca ion channel. * indicated that the *p*-value is 0.05 or less compared to the WT group. ** indicated that the *p*-value is 0.05 or less compared to the Vehicle-treated C3 KO group. Abbreviations: C3 KO, Complement component 3 knockout; Urd, Uridine; AELP, Aqueous extract of *Liriope platyphylla* L.; cAMP, Cyclic adenosine triphosphate; PKA, Protein kinase A; CFTR, Cystic fibrosis transmembrane conductance regulator; CLCN2, Chloride voltage-gated channel; SCN5A, Sodium voltage-gated channel alpha subunit 5; KCNQ, Potassium channel; CACNA1C, Calcium voltage-gated channel subunit alpha1 C; RT-qPCR, Quantitative real-time PCR; WT: Wild type.

Finally, we tried to determine the cause of the differences in the therapeutic effectiveness of Urd and AELP in the two models. To achieve this, alterations in the key parameters for the cyclic adenosine monophosphate (cAMP) downstream signaling pathway were analyzed in the mid-colons of the C3 KO and Lop-induced constipation models because it is commonly involved in the downstream signaling pathway of the C3 receptor and Lop binding opioid receptor [24,25] (Figure 8A and Table S1). The cAMP concentration showed reverse regulation patterns, which increased by 36% in C3 KO mice and decreased by 22% in the Lop-induced constipation model. These levels were differentially improved in the C3 KO (45 and 58%) and Lop-induced constipation models (48 and 32%) after the administration of Urd and AELP. Their effectiveness was greater in the C3 KO mice model than in the Lop-induced constipation mice model (Figure 8B). Also, similar alteration patterns were detected in the levels of p-PKA, a key member of the cAMP downstream signaling pathway. These levels were enhanced in the C3 KO and Lop-induced constipation models after administration of only Urd but not AELP (Figure 8C). Furthermore, the transcription levels of four cAMP-controlled ion channels, including Cl, Na, Ca, and K, were analyzed as ion imbalances and are considered one of the main causes of constipation [26]. The transcription levels of most ion channels except those of the K ion were lower in the Vehicle-treated mice with constipation compared to the control group regardless of the type of model (Figure 8D–F). However, those of the calcium voltage-gated channel subunit alpha1 C (CACNA1C) channel for the K ion showed a reverse pattern with an increase in the Vehicle-treated C3 KO and Lop-induced mice (Figure 8G). After the administration of Urd and AELP, they were significantly improved in both models. However, the therapeutic effectiveness of Urd and AELP varied in each treatment condition and model. The effectiveness of Urd and AELP on the cystic fibrosis transmembrane conductance regulator (CFTR) transcription for the Cl ion channel was detected in only the Lop-induced constipation model and not in the C3 KO model. Those of another Cl ion channel (chloride channel protein 2, CLCN2) showed a reverse pattern during constipation, although treatment of Urd and AELP were improved. Therefore, the effectiveness of the two therapeutics on CLCN2 transcription was difficult to compare (Figure 8D and Table 8). In the case of sodium channel protein type 5 subunit alpha (SCN5A) for the Na ion channel, the effectiveness of Urd and AELP was greater in the C3 KO model than in the Lop-induced constipation model (Figure 8E and Table 8). The effectiveness of Urd for the potassium channel KCNQ was higher in the Lop-induced constipation model than in the C3 KO model, while the AELP-treated groups showed a constant level (Figure 8F and Table 8). Moreover, the transcription of CACNA1C in the Urd-treated C3 KO model was higher than in the Lop-induced constipation model, while, after treatment with AELP, the transcription of CACNA1C was greater in the Lop-induced constipation model than in the C3 KO model (Figure 8G and Table 8). Therefore, the above results show that differences in the therapeutic effectiveness of Urd and AELP between C3 KO and Lop-induced constipation models may be associated with alterations of the cAMP downstream signaling pathway.

## 3. Discussion

The effectiveness of clinical management against most diseases depends on the intrinsic efficacy and system-dependent parameters, such as the density of receptors and the efficiency of receptor–effector coupling [27]. Therefore, to determine the efficacy of an agent against the target disease it is essential to compare its efficacy using animal models of different etiologies [28]. In this study, we compared the effectiveness of two therapeutic agents for constipation in two animal models with different etiologies to provide scientific evidence on their therapeutic target and mechanism of action. Our results suggest that Urd may be effective against C3-deficiency-induced constipation and AELP may be more effective against Lop-induced constipation, although further studies are needed to investigate the clear reasons for this difference.

In this study, we compared the therapeutic effectiveness of Urd and AELP on GI transit in C3 KO and Lop-induced constipation models. The GI transit ratio significantly improved in both models after the administration of Urd and AELP. These effects of Urd and AELP were very similar to those reported in previous studies, although the species used to create the Lop-induced constipation model was different [22,29]. The therapeutic effectiveness of Urd and AELP on the delay in GI transit was greater in the C3 KO than in the Lop-induced constipation model as shown in Figure 2. While the actual reason has not been accurately identified, this difference is likely to be due to several reasons such as the strategy of inducing constipation, the age of the experimental animals, and the animal species used to create the model. Few scientific studies provide clues about the link between GI transit and complement components. C3a, C3b, and their receptor signaling pathways are associated with the regulation of neuronal processes including neurogenesis after stroke, development of immature neuronal progenitors, migration of hippocampal neurons [30,31,32]. Therefore, further studies are needed on the action mechanism of the C3 proteins and their fragments on intestinal motility through the regulation of nerve cells based on these clues relating to C3 functions.

The disruption of water retention capacity is considered one of the major causes of constipation [33]. As the stool moves slowly through the large intestine, additional water is reabsorbed, resulting in the stool becoming hard, dry, and facing difficulty in moving through the lower intestine [34]. In the colon, water, and electrolytes are regulated by hormones such as aldosterone, glucocorticoids, and vasoactive intestinal peptides as well as the neuroendocrine mechanisms [35]. Also, the transmembrane AQPs play a key role in the regulation of water permeability and stimulation of water and electrolyte secretion in the colon via numerous signaling pathways [36]. Therefore, in this study, we compared the therapeutic effectiveness of Urd and AELP on the downregulation of AQPs expression between the C3 KO and Lop-induced constipation models. This effectiveness was much higher in the C3 KO group than in the Lop-induced constipation model. Also, the therapeutic effectiveness of Urd and AELP in the C3 KO constipation model was very similar to those of previous studies, but they were first presented in Lop-induced mice, not rats [18,22,23]. Therefore, our study provides the first evidence that the expression of AQPs for water retention capacity may tightly link to the cause of constipation.

Meanwhile, our results showed that the administration of Urd and AELP can improve the structure and function of the ENS in C3 KO and Lop-induced constipation models. Specifically, the effectiveness was analyzed with respect to the distribution of the neuronal cells and ICC, inhibitory function, and the excitatory function of the ENS, as shown in Figure 7. These results for Urd and AELP with respect to the improvement in the symptoms of constipation were similar to those seen in earlier studies using the C3 KO mice model, while those observed in Lop-induced constipation in ICR mice model were very similar to those using SD rats [18,22,23]. However, the therapeutic effectiveness of Urd and AELP was greater in the C3 KO model than in the Lop-induced model. The reason for this difference in therapeutic effectiveness can be found in indirect scientific evidence of the interrelationship between neuronal cells and complements. The complement system plays an important role in neuronal development and neurodegeneration [37]. Among them, C3 plays a role in progenitor proliferation, neuronal migration, and synaptic pruning in astrocytes, microglia, and neurons during normal development of the nerve system [38,39,40,41,42,43]. However, the detailed mechanisms of the effects of C3 proteins and their fragments on the inhibitory and excitatory function of the ENS have never been investigated to date. Therefore, our research results additionally provide scientific evidence that C3 proteins can be considered an important target in the treatment of constipation.

Meanwhile, our study indicates that the difference in the therapeutic effectiveness of Urd and AELP in C3 KO and Lop-induced constipation models could be associated with the cAMP downstream signaling mechanism because this pathway involves the binding of Lop and the opioid receptors as well as C3a binds to its receptor [20,21]. In this study, the cAMP levels were increased in the C3 KO and decreased in Lop-induced constipation models. These alterations of cAMP were reflected in the transcriptional level of only CLCN2 and KCNQ in C3 KO mice and only p-PKA and four ion channels in the Lop-induced model as a member of cAMP downstream signaling pathway. However, the expression level of p-PKA and other three ions channels was reversely decreased in C3 KO mice, as well as that of KCNQ was increased in Lop-induced model. After treatment of Urd and AELP, these levels were improved regardless of their type. In particular, the expression level of only CLCN2 was completely reflected the concentration of cAMP in C3 KO mice and Lop-induced model. This channel is well known as a target of lubiprostone for treating constipation through the activation of the chloride channel and increase in fluid excretion [44]. Our results provide the first evidence that C3 deficiency can be considered to be a cause for the upregulation of CLCN2, unlike their levels in Lop-induced constipation.

## 4. Materials and Methods

### 4.1. Preparation of Urd and AELP

Urd and AELP were prepared following the procedure detailed in a previous study [29,45]. Urd was purchased from Sigma-Aldrich Co. (Saint Louis, MO, USA). Plant materials of *Liriope platyphylla* L. were collected in June 2011 from a farm in the Miryang region, Republic of Korea, and deposited as a voucher specimen (WPC-11-010) in the Functional Materials Bank of the Pusan National University (PNU)—Wellbeing RIS Center, as described in a previous study [29]. After drying the roots of *Liriope platyphylla* L. at 60 °C, they were blended using an electric blender and subsequently extracted with distilled water (dH_2_O) (600 g:2 L ratio). This water extract was purified with circulating extraction equipment (IKA Labortechnik, Staufen, Germany) at 100 °C for 2 h. Finally, the purified solution was concentrated into dry pellets using a rotary evaporator (EYELA, Tokyo, Japan) and stored at −80 °C.

### 4.2. Care and Use of Animals

The animal protocol for comparative study on the laxative effects was reviewed and approved by the PNU-Institutional Animal Care and Use Committee (IACUC) (approval number: PNU-2023-0098). All mice were carefully managed in the PNU-Laboratory Animal Resources Center, which was accredited by the Korea Food and Drug Administration (KFDA, Cheongju, Republic of Korea) (accredited unit number: 000231) and the Association for Assessment and Accreditation of Laboratory Animal Care (AAALAC, Frederick, USA) International (accredited number: 001525). This facility was maintained under the specific pathogen-free (SPF) conditions, a straight light and dark cycle (on 8:00–20:00; off 20:00–7:00), a constant temperature (23 ± 2 °C), and relative humidity (50 ± 10%). A standard chow diet (Samtako BioKorea Co., Osan, Republic of Korea) and filtered tap water provided the mice ad libitum. Seven-week-old ICR mice as Lop-induced constipation model were supplied from Samtako BioKorea, while C3 KO mice (FVB/N-C3^em1Hlee^/Korl) reported in a previous study were kindly provided by the Laboratory Animal Resources Bank in the National Institute of Food and Drug Safety Evaluation (NIFDS, Cheongju, Republic of Korea) [17].

### 4.3. Experimental Designs for Constipation Induction

C3-deficiency-induced constipation was observed spontaneously in C3 KO mice aged 16 weeks, as described in a previous study [20]. C3 KO mice (homogenous type) were produced by mating heterogenous type (HT) males and females with an FVB/N Korl background and subsequently was confirmed with genotyping using specific primers [7,20]. Briefly, sixteen-week-old WT mice (*n* = 7) and C3 KO mice (*n* = 21) were used for the oral administration of Urd and AELP. Mice in the C3 KO group were further subdivided into the C3 KO Vehicle-treated group (Vehicle-treated C3 KO group; *n* = 7), Urd-treated group (Urd-treated C3 KO group; *n* = 7), and AELP-treated group (AELP treated C3 KO group; *n* = 7). The Urd- and AELP-treated C3 KO groups were orally administrated with the same volume (0.3 mL of dH_2_O) of 100 mg/kg and 1,000 mg/kg of Urd and AELP at one time, respectively, while the WT and Vehicle-treated C3 KO groups were orally administrated with only dH_2_O of the same volume. 

Constipation was induced using Lop (Sigma Aldrich Co., 4 mg/kg weight)in the Lop-induced constipation group, according to the method described in an earlier study [22]. Briefly, ICR mice (*n* = 28) were assigned to a non-constipation group (no group; *n* = 7) and a Lop-induced constipation group (Lop group; *n* = 21). The constipation group was subdivided into the Vehicle-treated group (Vehicle-treated Lop group; *n* = 7), Urd-treated group (Urd-treated Lop group; *n* = 7), and AELP-treated group (AELP-treated Lop group; *n* = 7). The Lop-induced constipation group was subcutaneously injected with Lop in 0.9% sodium chloride (DUKSAN Pure Chemicals Co., Ltd., Ansan, Republic of Korea), twice a day for 4 days. After 3 days for the stationary phase, 8 mg/kg Lop in 0.9% sodium chloride was subcutaneously injected for 4 days based on the methods described in a previous study [29]. After inducing constipation through Lop injection and C3 deficiency, each mouse orally received 0.3 mL of Urd (100 mg/kg) and AELP (1000 mg/kg). These dosages were referenced to a previous study that showed the optimal laxative effects of Urd and AELP in a Lop-induced model [22,23,45]. After collecting stools and urine samples from a metabolic cage, all mice of the subset groups (Lop-induced model and C3 deficiency model) were euthanized under the chamber with a gas regulator and CO_2_ gas with a minimum purity of 99.0% based on the American Veterinary Medical Association (AVMA, Schaumburg, IL, USA) Guidelines for the Euthanasia of Animals. Finally, the death of each mouse was verified by the absence of vital signs through ascertaining cardiac and respiratory arrest or dilated pupils and fixed bodies.

### 4.4. Analyses of the Stool Parameters

After collecting uncontaminated stools from a metabolic cage (Daejong Instrument Industry Co. Ltd., Seoul, Republic of Korea) individually, the stools-related parameters were analyzed as described in a previous study [17]. Total stools collected at 9 a.m. for two days were weighed twice using an electronic balance, and the number of them was counted twice. Also, their morphological features were evaluated using images taken with a digital camera. The water content of them was analyzed as follows:

Stool water content = (A − B)/A × 100

where the weight of fresh stools was indicated as A and the weight of the dried stools which were prepared at 60 °C for 12 h was indicated as B.

### 4.5. Analysis of GI Motility

The charcoal transit ratio in the mouse was analyzed with the method described in the previous studies [22]. Briefly, 0.5 mL of charcoal meal (Sigma-Aldrich Co.) (3% activated charcoal in 0.5% aqueous methylcellulose) was orally administrated into each mouse. After half an hour, mice were euthanized with CO_2_ gas under standard methods, and total GI tract was harvested from the abdomen of mice. The final ratio was calculated as follows:

Charcoal transit ratio (%) = [(Total intestine length − Transit distance of charcoal meal)/Total intestine length] × 100

### 4.6. Histopathological Analysis of the Mid-Colon

The mid-colons collected from each mouse were firstly fixed in 10% formalin solution (SM Medical Supply Co., Ltd., Seoul, Republic of Korea) for 48 h, embedded in paraffin wax (Leica Microsystems GmbH, Wetzlar, Germany), and sectioned into 4 μm thick slices on the slid glass. The tissue slices were stained with a H&E solution (Sigma-Aldrich Co.) under the established optimal conditions. After observing the morphological features of the mid-colon sections, the thickness of the mucosal layer and muscle layer in the mid-colon were measured using the Leica Application Suite (Leica Microsystems).

For mucin staining analyses, these sections were stained with an alcian blue staining solution (IHC world, Woodstock, MD, USA) in a similar method. The mucin intensity in the stained slices was measured using the Image J program 1.52a (NIH, Bethesda, MD, USA).

### 4.7. RT-qPCR Analysis

The total tissue RNAs were isolated from the mid-colon (30 mg) using RNAzol (Tet-Test Inc., Friendswood, TX, USA) as described in manufacture’s protocol. After determining the concentration of total RNAs using a NanoDrop Spectrophotometer (Allsheng, Hangzhou, China), complementary DNA (cDNA) of the mid-colon was synthesized with Superscript II (Thermo Fisher Scientific Inc., Waltham, MA, USA) under the optimal condition containing an oligo-dT primer (Thermo Fisher Scientific Inc.) and dNTP. And then, the specific DNA fragments of the target gene were amplified with 2 × Power SYBR Green (TOYOBO Co., Osaka, Japan) and a specific primer (Table S2) based on the cDNA template. The PCR program consisted of the following phases: denaturation at 95 °C for 15 s, annealing at 70 °C for 60 s, and extension at 70 °C for 60 s. And the reaction was carried out in 40 cycles. Finally, the threshold of fluorescence intensity was determined via a previous study [17].

### 4.8. Western Blot Analysis

Total tissue proteins were isolated from the mid-colon using a Pro-prep solution (Intron Biotechnology Inc., Seongnam, Republic of Korea) using the protocol suggested from the manufacturer and a previous study [18]. After measuring the concentration of total protein using a Bicinchoninic acid Protein Assay (BCA) kit (Thermo Fisher Scientific Inc.), the appropriate amount of protein (30 μg) was applied and run on 8–12% sodium dodecyl sulfate-polyacrylamide gel electrophoresis (SDS-PAGE) (Bio-Rad Laboratories, Inc., Hercules, CA, USA) for 2 h, and proteins with a specific range of sizes were transferred nitrocellulose membranes (Amersham^TM^ Protran^®^, Chicago, IL, USA) at 40 V for 2 h. And then, the specific proteins on the membranes were bound with the specific primary antibodies (Table S3) and sequentially the secondary antibody (HRP-conjugated goat anti-rabbit IgG, Cell signaling Tech., Danvers, MA, USA). Finally, the luminescence signals derived from each protein band on the membrane was detected using the FluorChem^®^ FC2 Imaging system (Alpha Innotech Co., San Leandro, CA, USA). The density of each band detected by the specific antibody was quantified using the AlphaView Program (Cell Biosciences Inc., Santa Clara, CA, USA).

### 4.9. Liquid Chromatography–Mass Spectrometry (LC–MS) Analysis

The concentration of cAMP in the mid-colon tissue was quantified using LC–MS (QTRAP^®^ 6500 model (Sciex, Concord, ON, Canada) coupled to a Waters ACQUITY UPLC I-Class Plus system (Waters Corp., Milford, MA, USA)). The ACQUITY UPLC HSS T3 column (Waters Corp., Milford, MA, USA; 2.1 × 50 mm, 1.8 µm particle size) was used for chromatographic separation at 40 °C. The mobile phases transported the samples using Solvent A (0.1% formic acid in water) and Solvent B (acetonitrile with 0.1% formic acid). Also, the gradient elution program at 0.4 mL/min of the flow rate involved following four processes: 0–2 min, 0% B; 2–7 min, linear gradient to 100% B; 7–7.5 min, hold at 100% B; and 7.5–10 min, re-equilibrate to 0% B after a 5 µL of sample injection. Furthermore, MS with a negative electrospray ionization (ESI-) mode was performed under the following condition: IonSpray Voltage: 4500 V; Source Temperature: 350 °C; Curtain Gas: 40 psi; Ion Source Gas 1 (GS1): 50 psi; Ion Source Gas 2 (GS2): 50 psi; and Collision Gas: MediumMultiple reaction monitoring (MRM) mode, which was used for detection. The transition for Multiple Reaction Monitoring (MRM) was performed at m/z 328.0 → 134.0 under a collision energy of −34 eV. The standard solutions of cAMP prepared in the same matrix were used as external calibration. The concentrations of cAMP in the tissue samples were calculated by interpolation based on the standard calibration curve.

### 4.10. Statistical Analysis

Sample size was determined based on previous studies [17,18]. Data normality (Shapiro–Wilk test) and homogeneity of variances (Levene’s test) were confirmed prior to one-way analysis of variance (ANOVA). Between-group differences were analyzed using one-way ANOVA in SPSS Statistics version 29; IBM Crop.: Armonk, NY, USA, 2022. Post-hoc analyses included Tukey’s test for multiple comparisons and Fisher’s exact test for categorical variables. Data are expressed as mean ± standard deviation (SD), and statistical significance was defined as *p* < 0.05. For multi-gene analyses, *p*-values were adjusted using the Benjamini–Hochberg false discovery rate (FDR).

## 5. Conclusions

In this study, we compared the therapeutic effectiveness of Urd and AELP in C3 KO and Lop-induced constipation models to investigate whether the efficacy of the clinical management used for constipation can be linked to the cause of this disease. These results show that the therapeutic effectiveness of Urd and AELP on the disruption of GI transit, water retention, and the ENS structure and function were higher in the C3 KO than in the Lop-induced constipation model. Also, these differences were reflected in the alteration in the levels of the cAMP downstream signaling pathway. Therefore, the overall results suggest that the differences in the therapeutic effectiveness of Urd and AELP between the two models may be linked tightly to the alternative regulation of the cAMP downstream signaling pathway. Furthermore, the results of the present study suggest that the cause of constipation should be considered in determining the therapeutic effectiveness of agents for symptomatic management. Especially, our finding provides important information about potential interactions, side effects, variability in composition, and efficacy of natural products that should be considered for clinical application. Nevertheless, our study had few limitations with respect to the molecular mechanism for therapeutics. The present study did not verify the pharmaceutical action of the therapeutics that were directly associated with the various causes of constipation as well as the correlation between cAMP and bioactive compounds in natural products. Also, this study has limitations on the clear recognition of the specificity of the constipation models and the lack of direct validation at the receptor level. Further studies will be needed to verify these effects in models with other causes of this condition.

## Figures and Tables

**Table 1:** Comparison of Urd and AELP effectiveness on the stool parameters between both mice models.

Change Ratio (%)	C3 KO			Lop		
	Vehicle	Urd	AELP	Vehicle	Urd	AELP
Stool number	−49 ± 9.5% ***	+128 ± 26.4% ***	+72 ± 9.3%	−35 ± 3.5%	+93 ± 2.7%	+128 ± 2.7% ***
Stool water contents	−57 ± 3.6%	+143 ± 13.7%	+319 ± 56.6% ***	−64 ± 4.5% ***	+601 ± 14.9% ***	+202 ± 23.7%

*** indicated the higher therapeutic effects among C3 KO mice and Lop-induced mice. Abbreviations: C3 KO, Complement component 3 knockout; Urd, Uridine; AELP, Aqueous extract of *Liriope platyphylla* L.; WT, Wild type.

**Table 2:** Comparison of Urd and AELP effectiveness on the GI transit and length between both mice models.

Change Ratio (%)	C3 KO			Lop		
	Vehicle	Urd	AELP	Vehicle	Urd	AELP
Transit ratio	−27 ± 6.3% ***	+40 ± 1.8% ***	+26 ± 4.4% ***	−10 ± 9.4%	+6 ± 19.5%	+11 ± 14.7%
Colon length	−18 ± 11.1% ***	+33 ± 13.6% ***	+12 ± 16% ***	−7 ± 10.3%	−1.8 ± 3.2%	+9 ± 8.5%
Total intestine length	+2 ± 2.4% ***	+2 ± 4.5%	+3 ± 5.8%	+0.5 ± 1.7%	−6 ± 2.2% ***	−8 ± 3% ***

*** indicated the higher therapeutic effects among C3 KO mice and Lop-induced mice. Abbreviations: C3 KO, Complement component 3 knockout; Urd, Uridine; AELP, Aqueous extract of *Liriope platyphylla* L.; Lop, Loperamide.

**Table 3:** Comparison of Urd and AELP effectiveness on the histology of the mid-colon between both mice models.

Change Ratio (%)	C3 KO			Lop		
	Vehicle	Urd	AELP	Vehicle	Urd	AELP
Mucosal layer	−44 ± 8%	+42.1 ± 7%	+67.3 ± 12.6%	−59 ± 3.8% ***	+124 ± 16.5% ***	+80 ± 6.3% ***
Muscle layer	−63.8 ± 2.1%	+17.4 ± 11.8%	+31.9 ± 8.6%	−66 ± 2.8% ***	+55 ± 9.5% ***	+53 ± 14.6% ***

*** indicated the higher therapeutic effects among C3 KO mice and Lop-induced mice. Abbreviations: C3 KO, Complement component 3 knockout; Urd, Uridine; AELP, Aqueous extract of *Liriope platyphylla* L.; H&E, Hematoxylin and Eosin; Lop, Loperamide.

**Table 4:** Comparison of Urd and AELP effectiveness on the junctional complex between both mice models.

Change Ratio (%)	C3 KO			Lop		
	Vehicle	Urd	AELP	Vehicle	Urd	AELP
Claudin-1	−60 ± 0.8%	+466 ± 54.1% ***	+542 ± 86.5% ***	−66 ± 13.9% ***	+36.9 ± 36.8%	+278 ± 45.3%
Occludin	−50 ± 5.2%	+28 ± 14.2% ***	−0.5 ± 8.6%	−57 ± 6.7% ***	−4 ± 16.8%	+75 ± 43.6% ***
ZO-1	−42 ± 4.6% ***	+71 ± 24.1% ***	−8.6 ± 5.3%	−25 ± 0.8%	−4 ± 11%	+75 ± 8.1% ***

*** indicated the higher therapeutic effects among C3 KO mice and Lop-induced mice. Abbreviations: C3 KO, Complement component 3 knockout; Urd, Uridine; AELP, Aqueous extract of *Liriope platyphylla* L.; WT, Wild type; Lop, Loperamide; ZO-1, Zonula Occludens-1.

**Table 5:** Comparison of Urd and AELP effectiveness on the mucin regulation between both mice models.

Change Ratio (%)	C3 KO			Lop		
	Vehicle	Urd	AELP	Vehicle	Urd	AELP
Mucin intensity	−49 ± 10.8%	+67 ± 15.4%	+61 ± 27.7%	−68 ± 4.3% ***	+319 ± 25.7% ***	+165 ± 11.6% ***
MUC2	−51 ± 7.8%	+156 ± 1% ***	+237 ± 21.9% ***	−73 ± 1.3% ***	+6 ± 9%	+130 ± 13.5%
MUC1	−25 ± 4.2%	+68 ± 7.3%	+222 ± 51% ***	−56 ± 6.7% ***	+245 ± 23.4% ***	+169 ± 7%
Klf4	−49 ± 10% ***	+92 ± 24.6% ***	+95 ± 26.7% ***	−41 ± 8%	+33 ± 8.4%	+0.9 ± 9.4%

*** indicated the higher therapeutic effects among C3 KO mice and Lop-induced mice. Abbreviations: C3 KO, Complement component 3 knockout; Urd, Uridine; AELP, Aqueous extract of *Liriope platyphylla* L.; WT, Wild type; MUC, mucin; Klf4, Krüppel-like factor 4.

**Table 6:** Comparison of Urd and AELP effectiveness on the AQP expression between both mice models.

Change Ratio (%)	C3 KO			Lop		
	Vehicle	Urd	AELP	Vehicle	Urd	AELP
AQP3	−55 ± 3.8% ***	+76 ± 29.9% ***	+476 ± 12.9% ***	−36 ± 10.3%	+9 ± 21.2%	+59 ± 17.8%
AQP8	−41 ± 11.3%	+400 ± 63.6% ***	+119 ± 58.7%	−63 ± 6.4% ***	+59 ± 20.4%	+270 ± 73.3% ***

*** indicated the higher therapeutic effects among C3 KO mice and Lop-induced mice. Abbreviations: C3 KO, Complement component 3 knockout; Urd, Uridine; AELP, Aqueous extract of *Liriope platyphylla* L.; AQP, Aquaporins.

**Table 7:** Comparison of Urd and AELP effectiveness on the ENS-related markers between both mice models.

Change Ratio (%)	C3 KO			Lop		
	Vehicle	Urd	AELP	Vehicle	Urd	AELP
c-kit	−14 ± 0.8%	+134 ± 2.3% ***	+12 ± 1%	−43 ± 0.8% ***	+113 ± 2%	+30 ± 1% ***
PGP9.5	−34 ± 1%	+0.8 ± 1.4%	+161 ± 2.2%	+23 ± 3.4%	−54 ± 2.3%	−20 ± 3.7%
NSE	−18 ± 3.4% ***	+12 ± 1.3% ***	+27 ± 2.3% ***	−10 ± 2.7%	+6 ± 0.8%	−18 ± 7.2%
5-HT 2AR	−32 ± 3.2%	+47 ± 12.2%	+365 ± 142.1% ***	−56 ± 14.2% ***	+250 ± 46.6% ***	+142 ± 12.9%
5-HT 2BR	−62 ± 7.5% ***	+36 ± 25.5% ***	+65 ± 17.1% ***	−43 ± 2.7%	+28 ± 20%	+0.3 ± 18.4%
5-HT 3AR	−64 ± 4.5% ***	+179 ± 19.5% ***	+203 ± 38.2% ***	−16 ± 1.9%	−48 ± 7.3%	+104 ± 40.9%
5-HT 3BR	−81 ± 7%	+890 ± 72%	+637 ± 182.8%	+64 ± 11.6%	−46 ± 4% ***	−24 ± 0.3% ***
mAChR M3	−9 ± 2%	+4 ± 0.8%	+13 ± 2.8% ***	−44 ± 1.6% ***	+9 ± 1.2% ***	+5 ± 1.6%
mAChR M2	−15 ± 4.4% ***	+6 ± 4.9% ***	+35 ± 7.4% ***	−12 ± 0.8%	+4 ± 1.6%	+5 ± 1.8%
Gα	+47 ± 13% ***	−19 ± 6.8% ***	−39 ± 7%	+43 ± 1.8%	−5 ± 1.4%	−58 ± 0.3% ***
Phosphorylation of PI3K	+127 ± 53.5% ***	−19 ± 19.1% ***	−43.1 ± 11.1% ***	+50 ± 15.2%	−6.3 ± 9.7%	−9 ± 11%
Phosphorylation of PKC	+37 ± 4.1%	−32 ± 1% ***	−22 ± 4.3% ***	+117 ± 6.9% ***	−21 ± 6.5%	−3 ± 7.8%

*** indicated the higher therapeutic effects among C3 KO mice and Lop-induced mice. Abbreviations: C3 KO, Complement component 3 knockout; WT, Wild type; Urd, Uridine; AELP, Aqueous extract of *Liriope platyphylla* L.; C-kit, Receptor protein kinase kit; NSE, Neuron-specific enolase; PGP9.5, Protein gene product 9.5; 5-HT, 5-hydroxytryptamine; mAChRs, Muscarinic ac-etylcholine receptors; ENS, Enteric nervous system; Lop, Loperamide.

**Table 8:** Comparison of Urd and AELP effectiveness on the cAMP downstream signaling pathway between both mice.

Change Ratio (%)	C3 KO			Lop		
	Vehicle	Urd	AELP	Vehicle	Urd	AELP
cAMP	+36 ± 5.8%	−45 ± 2.5%	−58 ± 3.7%	−22 ± 4.8%	+48 ± 13.4%	+32 ± 3.9%
p-PKA	−27 ± 3.1%	+18 ± 3.8%	+8.4 ± 0.8% ***	−44 ± 0.7% ***	+20 ± 2.4% ***	−39 ± 0.5%
CFTR	−58 ± 9.1%	−64 ± 10.9%	−16 ± 21.3%	−68 ± 10.2% ***	+185 ± 52.9% ***	+70 ± 13.5% ***
CLCn−2	+33 ± 13.6%	−38 ± 3.6%	−64 ± 6.4%	−73 ± 9.7%	+19 ± 28.3%	+103 ± 20.5%
NaV1.5	−35 ± 9.2%	+85 ± 4.1% ***	+435 ± 32.4% ***	−54 ± 7% ***	+48 ± 11.2%	+121 ± 5.5%
KV1.2	+144 ± 18.4%	−36 ± 10.9%	−55 ± 7.5%	+161 ± 7.3% ***	−74 ± 2.2% ***	−59 ± 3.8% ***
CaV1.2	−39 ± 7.5%	+70 ± 23.1% ***	+0.5 ± 18.3%	−51 ± 5.8% ***	+9 ± 23.3%	+44 ± 11.1% ***

*** indicated the higher therapeutic effects among C3 KO mice and Lop-induced mice. Abbreviations: C3 KO, Complement component 3 knockout; Urd, Uridine; AELP, Aqueous extract of *Liriope platyphylla* L.; cAMP, Cyclic adenosine triphosphate; PKA, Protein kinase A; CFTR, Cystic fibrosis transmembrane conductance regulator; CLCN2, Chloride voltage-gated channel; SCN5A, Sodium voltage-gated channel alpha subunit 5; KCNQ, Potassium channel; CACNA1C, Calcium voltage-gated channel subunit alpha1 C.

## Data Availability

Data are contained within the article.

## Supplementary Materials

The following supporting information can be downloaded at https://www.mdpi.com/article/10.3390/ph18091289/s1. Table S1. Proposed constipation-inducing mechanism in C3 KO and Lop-induced mice; Table S2. Primer sequence for RT-qPCR analyses; Table S3. Antibody list for western blot analyses.

## Abbreviations

The following abbreviations are used in this manuscript:

Lop	Loperamide
C3	Complement component 3
AELP	Aqueous extract of *Liriope platyphylla* L.
Urd	Uridine
ENS	Enteric nervous system
GI	Gastrointestinal
cAMP	Cyclic adenosine monophosphate
KO	Knock out
WT	Wild type
SD	Sprague-Dawley
H&E	Hematoxylin and eosin
TJ	Tight junction
ZO-1	Zonula occludens-1
MUC	Mucin
Klf4	Krüppel-like factor 4
AQP	Aquaporins
ICC	Interstitial cells of Cajal
C-kit	Receptor tyrosine kinase protein
PGP9.5	Protein gene product 9.5
NSE	Neuron-specific enolase
HT	5-hydroxytryptamine
mAChRs	Muscarinic acetylcholine receptors
CLCN2	Chloride voltage-gated channel
SCN5A	Sodium channel protein type 5 subunit alpha
CACNA1C	Calcium voltage-gated channel subunit alpha1 C
