# Supplementary material for: Differences in the Effectiveness of Uridine and Liriope platyphylla L. Between Complement Component 3 Deficiency- and Loperamide-Induced Constipation May Be Associated with the Alternative Regulation of the Cyclic Adenosine Monophosphate Downstream Signaling Pathway
Source: Pharmaceuticals (Basel). 2025 Aug 28;18(9):1289. doi: 10.3390/ph18091289 (PMC12473127; doi:10.3390/ph18091289)
Supplement: Supplementary file 1 [file pharmaceuticals-18-01289-s001.zip › pharmaceuticals-3801133-supplementary.pdf]

Supplement Table S1. Proposed constipation-inducing mechanism in C3 KO and Lop-induced mice

Classification	Cause	Proposed action mechanism	References
Loperamide induced constipation	Intraperitoneal injection of loperamide	-Inhibit the release of ACh from neuronal cells and reduce intestinal motility through binding to opioid receptors.<br>-The binding of loperamide and opioid receptor induce the suppression of cAMP signaling pathway, and increasing the absorption of ions such as moisture/electrolytes. And then it causes to the decreasing the relaxation of smooth muscles.	Imam et al., 2018<br>Li et al., 2023
C3 deficiency induced constipation	Knockout of Complement C3 gene	-The exact mechanism of action has not yet been reported.<br>-May associate with the dysregulation of C3 receptor downstream signaling pathway (involvement of cAMP)<br>-May associate with the dysregulation of the enteric nervous system (distribution and function).<br>-May associate with the promotion of the inflammatory response.<br>-May associate with the inhibition of gastrointestinal transit and mucin secretion.	Park et al., 2019<br>Choi et al., 2022a<br>Choi et al., 2022b

Supplementary Table S2. Primer sequence for RT-qPCR analyses

Gene name	Sequence (from 5' to 3')
ZO-1	
Forward	CCTCC GTTGC CCTCA CAGTA
Reverse	GGGCG CCCTT GGAAT G
Claudin-1	
Forward	CCCCG GAAAA CAACC TCTTA C
Reverse	TGTCA CACAT AGTCT TTCCC ACTAG AA
Occludin	
Forward	TTGAA GAGTG GGTTA AAAAT GTGTC T
Reverse	TCAAC TCTTT CCGCA TAGTC AGAT
MUC2	
Forward	GCA CAT TCC TTC GCA TCT TAA A
Reverse	AAA GCA AAG AAT GGA ACA GAA CAG AAA CTC
MUC1	
Forward	CGCCA GCCTT GAGTT TGTTT
Reverse	GAAGA AAGGA GCCCG AATGC
Klf4	
Forward	GGTGC AGCTT GCAGC AGTAA
Reverse	AAGTC TAGGT CCAGG AGGTC GTT
AQP3	

Forward	GGTGG TCCTG GTCAT TGGAA
Reverse	AGTCA CGGGC AGGGT TGA
AQP8	
Forward	TCGCT GGCAG TCACA GTGA
Reverse	TCCAA ATAGC TGGGA GATCC A
5-HT2AR	
Forward	CCGGG AGCCT CTTGA TACAG
Reverse	AGCCC CTCTC AAAGT CACAC A
5-HT2BR	
Forward	GCAGA TTTGC TGGTT GGATT G
Reverse	GGCCA TATAG CCTCA AACAT GAT
5-HT3AR	
Forward	CTGAG GCCCT CCCAC ATCT
Reverse	GGAAA GGAAC AAGGC CAACA
5-HT3BR	
Forward	TGCCG AGGAG TCTAG ATTGT ACCT
Reverse	ACCCG ATGCT CCTGA TGGA
CFTR	
Forward	TCTGC CGCGC AGCAA
Reverse	GGTGT GAACG TCATC AGATC CA
CLCn-2	
Forward	CAGCA CATGC AAAAG CTAAG AAAA
Reverse	GCGGA TAGAT GTCTC GGAGC TA
SCN5A	
Forward	CGGTC CCAGA GCATT GAATC
Reverse	CTGTA CTGTT CTCGT CATCT GCAA
KCNQ	
Forward	GGGCA CACAA GGCAA CATCT
Reverse	CAGGA TCTGT AGGAA GCGCA TA
CACNA1C	
Forward	TACAG CCTTC AAATG TGGTC
Reverse	TCTGA CTTCA CAACT GAACA
β-actin	
Forward	TGGAA TCCTG TGGCA TCCAT GAAAC
Reverse	TAAAA CGCAG CTCAG TAACA GTCCG

Supplementary Table S3. Antibody list for western blot analyses

**Antibody**	**Company**	**Catalog number**
Anti-c-kit	Abcam Com., Cambridge, UK	Ab256345
Anti-PGP9.5	Abcam Com., Cambridge, UK	Ab108986
Anti-NSE	Abcam Com., Cambridge, UK	Ab180943
Anti-mAChR M3	Alomone Labs, Jerusalem, Israel	AMR-006
Anti-mAChR M2	Alomone Labs, Jerusalem, Israel	AMR-002
Anti-Gα	Cell Signaling Technology Inc., Cambridge, MA, USA	#5290
Anti-PI3K	Cell Signaling Technology Inc., Cambridge, MA, USA	#4292
Anti-p-PI3K	Cell Signaling Technology Inc., Cambridge, MA, USA	#4228
Anti-PKC	Cell Signaling Technology Inc., Cambridge, MA, USA	#2056

Anti-p-PKC	Cell Signaling Technology Inc., Cambridge, MA, USA	#9375
Anti-p-PKA	Cell Signaling Technology Inc., Cambridge, MA, USA	#9621
Anti-β-actin	Cell Signaling Technology Inc., Cambridge, MA, USA	#4967
